# Qualitative Research Leaders: Evaluation of Pilot Global Pipeline Program

**DOI:** 10.4269/ajtmh.24-0381

**Published:** 2024-12-10

**Authors:** Monique M. Hennink, Donna J. Ingles, Blandina T. Mmbaga, Dorairaj Prabhakaran, Douglas C. Heimburger, Leslie C. M. Johnson

**Affiliations:** ^1^ Hubert Department of Global Health, Rollins School of Public Health, Emory University, Atlanta, Georgia;; ^2^ Center for Technology Transfer and Commercialization, Vanderbilt University, Nashville, Tennessee;; ^3^ Kilimanjaro Clinical Research Institute, Kilimanjaro Christian Medical Centre and Kilimanjaro Christian Medical University College, Moshi, Tanzania;; ^4^ Public Health Foundation of India and Centre for Chronic Disease Control, New Delhi, India;; ^5^ Vanderbilt Institute for Global Health, Vanderbilt University Medical Center, Nashville, Tennessee;; ^6^ Department of Family and Preventive Medicine, School of Medicine, Emory University, Atlanta, Georgia

## Abstract

Qualitative research methods are central to understanding many public health problems. However, capacity building for qualitative research is globally skewed toward high-income countries, with a significant skills deficit in low- and middle-income countries (LMICs). To address this imbalance and provide a model program, we developed the Qualitative Research Leaders (QRL) program, a pipeline program to increase qualitative research capacity in LMIC institutions and foster leadership in qualitative research. The QRL program is a collaboration between Emory University and Vanderbilt University in the United States and two LMIC institutions Kilimanjaro Christian Medical College in Tanzania and the Public Health Foundation of India. The program had five phases conducted over 12 months: 1) mentored study design sessions, 2) an in-country skill-building workshop, 3) mentored proposal development sessions, 4) a residential grant writing program in the United States, and 5) mentor matching for field implementation. Program evaluation results showed that the QRL program was effectively designed, and scholars valued the program components, learning formats, and session content. The program increased scholars’ knowledge, skills, and confidence as mentors in qualitative research. Furthermore, scholars demonstrated leadership in initiating postprogram research, mentoring, and teaching in qualitative research. Overall, scholars felt that the program could be extended to enable more time to internalize new knowledge and develop skills, and the role of in-country mentors could be expanded to further support scholars during fieldwork. In this article, we describe the components of the QRL program and its implementation, results of our program evaluation, and lessons learned for developing similar pipeline training programs.

## INTRODUCTION

The growing importance of qualitative research is reflected in its increasing demand among researchers in public health and medicine. Qualitative research methods courses are now part of the curricula across a diverse range of disciplines, and qualitative research skills are now a required competency for all Council For Education on Public Health (CEPH)-accredited schools of public health in the United States.^1^ The increase in mixed methods research across the health sciences has also created greater demand for learning qualitative research. Despite clear recognition of the importance of qualitative methods for public health research, there are significant disparities in training opportunities globally, which are skewed toward high-income countries (HICs).^2^ Conversely, capacity building for qualitative research has lagged in low- and middle-income countries (LMICs), creating a significant deficit in skills and expertise in these countries.

The lack of training opportunities means that investigators from LMICs have limited expertise in writing effective research proposals for qualitative or mixed methods research, curtailing their success in receiving research funding.^3^ The resulting lack of expertise means that LMIC investigators often do not use qualitative methods, even if they are most appropriate for a study. Qualitative research in LMICs is often led by scientists from the United States and other HICs, further perpetuating the capacity gap.^3^ This capacity gap is likely to widen if training opportunities for LMIC investigators in qualitative research are not created, as new required competencies for CEPH accreditation in the United States will create a much larger pool of US-based public health scholars trained in qualitative research.^1^^,^^3^

The critical shortage of LMIC investigators trained in qualitative research is problematic. The use of qualitative methods and their integration into quantitative studies requires careful application of core principles to maintain scientific rigor.^3^^–^^5^ There is a strong concern about the quality of qualitative research undertaken without robust training, as researchers may opt for or be encouraged to conduct qualitative research without substantive training. Poor-quality qualitative research wastes scarce resources, reduces the credibility of qualitative research, and may compromise the validity of the research findings that result.^2^^,^^6^ This situation underscores a significant and urgent need to build capacity among LMIC investigators to conduct robust qualitative research and mentor others in their field locally.

We sought to address critical gaps in qualitative research capacity in LMIC institutions and develop a cadre of emerging leaders in qualitative research by designing and evaluating a pilot training program on qualitative research methods for LMIC investigators—the Qualitative Research Leaders (QRL) program. Here, we describe the components of the pilot program, its implementation, and the results of our evaluation of program components and identify lessons learned that may be beneficial for similar pipeline programs in LMICs.

## MATERIALS AND METHODS

### The QRL pilot program.

#### Partner institutions.

The QRL pilot program was an institutional collaboration between Emory University, Vanderbilt University, and two LMIC partner institutions the Kilimanjaro Christian Medical Centre in Tanzania and the Public Health Foundation of India. Emory University faculty led the QRL program, delivered the training workshop (QUAL-WORKS), and mentored QRL scholars. Vanderbilt University faculty contributed administrative and logistical support and conducted the well-established immersive grant writing training program (Vanderbilt Institute for Research Development and Ethics [VIRDE]^7^). The two LMIC partner institutions were selected due to their longstanding collaboration with the US institutions, their focus on public health research, and their readiness for capacity building in qualitative research as a priority need for research staff. Both institutions are culturally and geographically distinct, thus allowing us to assess the fit of the program within different global contexts. LMIC institutions provided logistical support to host the in-country training workshops, managed the program within their respective institutions, mentored scholars, and monitored deliverables.

The pilot phase of the QRL program was funded by the Fogarty International Center of the NIH as a supplement to the Vanderbilt–Emory–Cornell–Duke (VECD) Consortium for Global Health Fellows program.

#### QRL program leadership.

The program leadership comprised senior faculty from Vanderbilt University Medical Center and Emory University Rollins School of Public Health, LMIC site directors, and a VECD steering committee. The steering committee—faculty from the VECD program with extensive experience in research training and mentoring in LMIC settings—provided guidance as needed.

#### Scholar selection.

To recruit high-caliber QRL scholars, applicants were required to hold a doctoral-level degree, have held a research position in an LMIC partner institution for at least 1–2 years, be able to fully participate in all program phases, be able to travel to the United States for the month-long immersive phase, have strong institutional support via letters of recommendation, and have demonstrated the ability to conduct health-related research. The QRL program was advertised internally at the partner institutions, and scholars applied through a website portal. Candidates were selected through a competitive process whereby applications were reviewed and scored independently by two reviewers within the QRL program leadership. Applications were then ranked by institution, and the highest-ranking applications at each institution were considered. LMIC site directors provided input on the final pool of candidates. At each step, reviewers aimed to ensure that the applicant pool was diverse in terms of sex and career level. Four QRL scholars were selected to participate in the program.

#### Program goals.

Our goal was to design, pilot, and evaluate a training pipeline to reduce the capacity gap in qualitative research in LMIC public health institutions that could be replicated in other LMIC settings and to foster a cadre of emerging leaders in qualitative research. The following were the specific aims of the program:

1. To design a training pipeline to facilitate leadership in qualitative research in LMIC institutions.
2. To foster a supportive environment for qualitative research within participating institutions.
3. To evaluate core components of the QRL program using mixed methods.

#### Program design and implementation.

**Figure 1.**
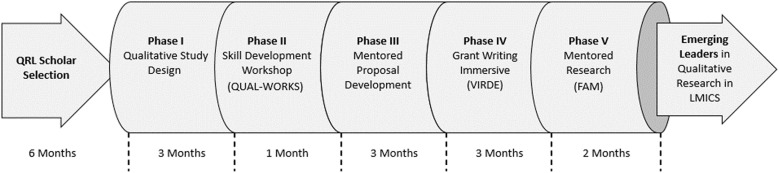
QRL program phases. FAM = Fellow–Alumni–Mentor; LMICS = low- and middle-income countries; QRL = Qualitative Research Leaders; VIRDE = Vanderbilt Institute for Research Development and Ethics.

The QRL program began in mid-2019 and consisted of five interlinked phases (shown in Figure 1). It was designed to be delivered over 12 months, preceded by a 6-month planning phase that included course development and scholar selection, and followed by a 12-month postprogram evaluation. However, program timing was interrupted by the coronavirus disease 2019 (COVID-19) pandemic, so final program evaluations were completed in 2024. The QRL program linked together highly successful existing programs (QUAL-WORKS, VIRDE, and Fellow–Alumni–Mentor [FAM]) and supplemented them with intensive individual mentoring by world-class experts on qualitative research. QRL scholars progressed through the program to develop methodologically rigorous research proposals that could be submitted to a funding agency. The program format comprised asynchronous resources on Canvas, including all virtual lectures, reference material, and scholars’ submissions, and synchronous sessions via Zoom for interactive components, such as scholars’ presentations, discussions, and mentor feedback. Below, we describe the five phases of the QRL program as designed as well as several adjustments made due to the COVID-19 pandemic. A detailed description of each phase, including the goal, format, and outcomes for scholars, is presented in Table 1.

- **Phase I: Study design.** Phase I focused on scholars developing their ideas for a qualitative study. This phase comprised three virtual sessions conducted monthly to orient scholars to the QRL program, identify a clear research problem affecting a local community, and refine a research question or study objective suitable for qualitative research.
- **Phase II: Building skills and institutional support.** Phase II comprised a 5-day intensive training workshop (QUAL-WORKS^8^) on qualitative research that was conducted in-country at participating institutions in Tanzania and India. QUAL-WORKS is a training program for public health professionals on qualitative research developed at Emory University. Workshop participants included the QRL scholars plus a broader group of research faculty, staff, managers, and senior research directors from the same institution (*N* = 25). This fostered a supportive institutional environment for qualitative research by providing a greater understanding of qualitative and mixed method research (responding to our second aim).
- **Phase III: Mentored proposal development.** Phase III involved intense mentoring and methodological guidance on scholars’ research proposals. This phase comprised six virtual sessions conducted over 3 months that focused on writing NIH-style qualitative or mixed methods research proposals. Scholars also provided peer critiques of the draft proposals to develop confidence in critiquing and mentoring others on the methodological rigor of qualitative study design. Proposals were submitted to VIRDE for Phase IV.
- **Phase IV: Grant writing and submission.** Phase IV involved participation in a 1-month immersive grant writing program (VIRDE^7^) designed to support researchers in LMICs. This phase was directed by the Vanderbilt Institute for Global Health and was held at Vanderbilt University Medical Center in the United States. QRL scholars had reserved admission to the VIRDE program, but acceptance was dependent on successful review of their draft research proposals by the VIRDE Selection Committee. Although the VIRDE program provides instruction on the entire research process from seeking funding to project management, it does not provide a deep dive into qualitative methodology. Therefore, QRL scholars received mentoring specifically focused on qualitative methodology in Phase III as critical preparation for their VIRDE application, complementing other components of the VIRDE program. Phase IV lasted 3 months and included preparation time (e.g., travel logistics and visa application), VIRDE participation, and postprogram reflection on scholars’ research projects.
- **Phase V: Mentored research.** Phase V aimed to match scholars at the end of the QRL program with an experienced qualitative research mentor through the Fogarty FAM Network to provide ongoing mentorship as scholars secured funding and began the field implementation of their qualitative study (which may extend well beyond the QRL program). Over 2 months, QRL scholars worked independently with their mentors to develop a mentoring plan tailored to their specific project and individual needs using resources from the FAM Network website. We encouraged QRL scholars to join the FAM Network as mentors themselves in the future to foster a sustainable cadre of experts on qualitative research to support other scholars in LMIC institutions.

**Table 1:** Description of QRL program phases

Program Phase	Goal and Description	Format	Scholar’s Outcomes
**Phase I:** Study design	**Goal:** To develop ideas for a qualitative study Three introductory sessions oriented scholars to the QRL program and qualitative study design. Scholars reviewed asynchronous resources on qualitative study design and identified appropriate and contextually relevant qualitative or mixed methods research ideas. Mentors provided feedback via synchronous discussion sessions	**Virtual** Asynchronous resources and tasks Synchronous feedback	Know QRL program requirements Submit a workplan and joint agreement with institutional managers for protected time Identify a local research issue and research question/study objective
**Phase II:** Building skills and institutional support	**Goal 1:** To build qualitative research knowledge and skills The QUAL-WORKS workshop provided scholars with intensive training in qualitative research principles and skills. Modules were culturally adapted and composed of study design frameworks, data collection (recruitment, sample size, method selection, instrument design, and data collection), ethical issues, field team training, writing, and assessing qualitative research **Goal 2:** To build institutional support for qualitative research Research colleagues participated in QUAL-WORKS to foster a supportive environment for qualitative research in scholar’s institutions and initiate collaborations on mixed methods research	**In person** Group training	Know the principles of qualitative research Develop skills in qualitative data collection Foster a supportive environment for qualitative research within own institution Foster research collaborations with colleagues using qualitative/mixed methods
**Phase III:** Mentored proposal development	**Goal:** To develop a qualitative/mixed method research proposal Scholars received six sessions on developing a research proposal for qualitative/mixed methods research. Sessions aligned with sections of an NIH-style research proposal, including Specific Aims, Significance and Innovation, participant recruitment, data collection, and study timeline, with an emphasis on the qualitative methodology for each section. Scholars received individual feedback from mentors on each section alongside peer critiques	**Virtual** Asynchronous resources and tasks Synchronous feedback Peer critiques	Write draft qualitative research proposal and submit to VIRDE for Phase IV Develop confidence in mentoring others on qualitative research
**Phase IV:** Grant writing and submission	**Goal:** To develop grant writing skills and proposal submission Scholars participated in the VIRDE immersive program on grant writing and research management skills. Modules included research funding sources, grant writing strategies, research ethics, study design, scientific publishing, grants administration, budgets, mock grant review, academic leadership, and research project management. Scholars completed their research proposals, identified a funding source, and submitted their proposal	**In person** Group training Individual feedback from VIRDE mentors	Develop grant writing and research management skills Finalize research proposal Submit research proposal to a funding organization identified during VIRDE
**Phase V:** Mentored research	**Goal:** To receive ongoing mentorship in qualitative research Scholars are matched with a mentor in the FAM network to provide continuity of support after the QRL program. Mentors are selected by country/region, type of institution, language, career level, sex, and skills. Scholars mentoring relationships with FAM mentors enabled culturally relevant and topic-specific research guidance	**Virtual or in person** (based on mentor location) Individual mentoring	Identify a mentor in FAM network Develop a mentoring relationship with qualitative research experts Aim to join FAM as a future mentor

FAM = Fellow–Alumni–Mentor; QRL = Qualitative Research Leaders; VIRDE = Vanderbilt Institute for Research Development and Ethics.

#### Program adjustments (COVID-19 pandemic).

Phase II of the QRL program began in March 2020 and was impacted by the COVID-19 pandemic, which necessitated adjustments and flexibility due to global shutdowns and extended travel restrictions. Despite challenges and adjustments, the program was able to meet its overarching goals. The beginning of the pandemic necessitated pausing the QRL program for several months; when it resumed, the scholars in India were no longer employed at their institution, so new scholars were recruited. This required sessions in Phase I to be compressed and reordered to quickly integrate two new scholars. The Phase II workshop was conducted in person in Tanzania for QRL scholars and selected colleagues but was delivered online for India QRL scholars due to travel restrictions, although the workshop content was identical. During Phase III, all scholars received intensive mentoring to develop their research proposals; however, scholars in Tanzania received initial mentored sessions in a more compressed and less structured format to target a grant deadline, whereas scholars in India followed the original timeline. This flexibility often mirrors the reality of research grant application submission processes. During Phase V, FAM matching was replaced with continued guidance from a team of mentors, including QRL mentors, in-country mentors, and other subject matter experts. This provided continuity of mentorship for scholars and circumvented availability issues of FAM mentors during the pandemic. The core QRL program was designed to be delivered in 12 months; however, its implementation amidst the COVID-19 pandemic led the program to span 18 months. Two of the four scholars dropped out after completing Phase III due to new leadership responsibilities that arose due to the COVID-19 pandemic and related heavy workload issues. Thus, the remaining two scholars completed all program components.

#### Program evaluation.

The components of the QRL program were evaluated using mixed methods. The evaluation was designed to assess the impact of the QRL program on scholars’ knowledge, skills, and postprogram productivity in qualitative research (e.g., funding, publication, presentations, and mentoring provided). Scholars completed a brief survey after each phase of the program to evaluate the design, content, and delivery of program phases. In addition, pre- and postsurveys were used to assess changes in knowledge and skills improvement among all participants in the QUAL-WORKS workshop. Surveys were administered via Survey Monkey, and descriptive statistics were generated. To determine the longer-term impact of participating in the program, we conducted a 12-month postprogram in-depth interview and survey of scholarly productivity since entering the program with scholars who completed all program phases. In-depth interviews were conducted via Zoom by an independent interviewer not affiliated with the program, transcribed verbatim, and reviewed for key themes.

## RESULTS

### Evaluation results.

#### Phase I.

Phase I involved orienting scholars to the QRL program, developing an institutional workplan, and defining their study objectives. This phase was evaluated using a brief survey whereby scholars rated statements on a five-point scale from “strongly disagree” (1) to “neutral” (3) to “strongly agree” (5) and provided responses to open-ended questions. Scholars’ responses (Supplemental Figure 1) showed that they strongly agreed that developing a workplan was helpful to effectively participate in the program (score = 5) and agreed they were comfortable using the program learning platforms (Canvas [score = 4.75] and Zoom [score = 5]). However, scholars reported some challenges due to global time differences and internet connectivity, with sessions scheduled late at night or unstable internet connectivity causing disruptions to discussions and missed feedback. Scholars also stated that “course materials and constant feedback were very beneficial in improving the qualitative study.” Scholars strongly agreed that both the written and verbal feedback (both scores = 5) from mentors were useful in shaping their study objectives. Having both forms of feedback also helped where internet connectivity disrupted discussion-based feedback. In addition to mentor feedback, scholars valued interactive peer-to-peer feedback, for example, “The sessions were interactive. Teaming up with peers and getting feedback from them was helpful in shaping up the proposal and incorporating the discussions in the study.” Scholars reported confidence in critiquing a qualitative research proposal (score = 4.75). They also felt confident designing their research objectives (score = 4.5) and mentoring others (score = 4.25), although these scores were slightly lower. This may simply reflect that during Phase I, scholars had not yet given extensive critiques to others despite valuing this form of feedback.

#### Phase II.

**Figure 2.**
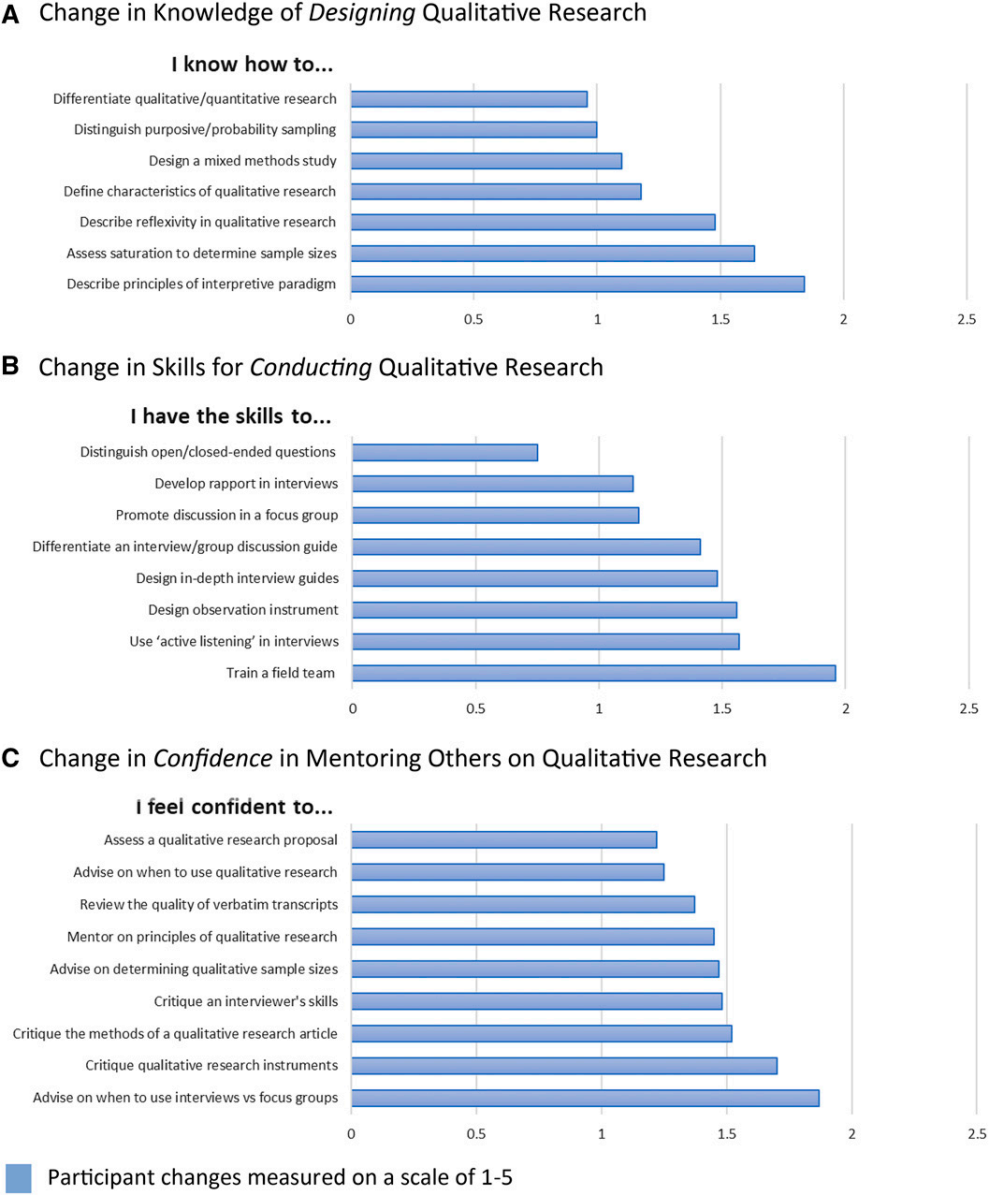
Changes in knowledge (**A**), skills (**B**), and confidence (**C**) from QUAL-WORKS Workshop (Phase II; *N* = 20). *Workshop participants included Tanzanian Qualitative Research Leaders scholars (*N* = 2) and 23 colleagues from Kilimanjaro Christian Medical Centre (total *N* = 25); however, 5 participants from Kilimanjaro Christian Medical Centre did not complete the postworkshop evaluation; thus, this figure represents data from 20 participants.

Phase II comprised a 5-day intensive training workshop that was evaluated using a pre–post survey of participants to assess changes after workshop participation. Participants were asked about their level of experience in qualitative and quantitative research before the workshop. The questions focused on three areas: knowledge of designing qualitative research (e.g., defining qualitative research, study design, and research concepts [e.g., saturation and reflexivity]), understanding skills for conducting qualitative research (e.g., instrument design, data collection skills, and field training), and confidence in mentoring in qualitative research (e.g., advising on study design/principles, assessing data collection skills, and critiquing qualitative research). Changes were measured on a five-point scale. We analyzed the survey results of QRL scholars and their colleagues separately; however, as the patterns of results were similar, we report the combined results. As expected, most participants had prior experience with designing and conducting quantitative research but little experience with qualitative research before the workshop. Few had experience with providing research mentoring, either in qualitative or quantitative research (data not shown). Figure 2A shows participants’ change in knowledge of designing qualitative research after the training workshop. Knowledge increased on all items assessed. The least change in knowledge was on how to distinguish qualitative and quantitative research, sampling approaches or design a mixed methods study. The highest changes were seen in participants’ understanding of the interpretive paradigm and concepts of reflexivity and saturation. Figure 2B shows changes in understanding of how to use specific skills for conducting qualitative research. Participants’ skills increased on all items measured. The lowest change was seen in distinguishing open- versus close-ended questions. All other skills increased at a similar level, such as instrument design, development of rapport, active listening, and promotion of discussion. In the open-ended responses, participants also highlighted the effectiveness of the skills-based activities for learning specific skills for qualitative research. Figure 2C shows changes in participants’ confidence for mentoring others in qualitative research. Participants’ confidence increased on all items. The highest increases in confidence also mirrored participation in specific skills-based activities conducted in the workshop, which enabled the application of specific principles and skill development (e.g., determining the appropriate use of qualitative methods, critiquing instruments, and assessing interviewers).

#### Phase III.

Phase III involved intense mentoring of scholars to develop their research proposals. This phase was evaluated using a short survey, whereby scholars rated statements related to their understanding, preparedness, and confidence in developing their research proposal as well as statements related to the mentoring approaches, resources, and learning platform used. The survey also included open-ended questions. Because of the pandemic, Phase III was accelerated and less structured for the Tanzanian scholars, which necessitated using different rating statements, therefore, the results are described separately.

Table 2 shows that Indian scholars reported mostly strong agreement with the usefulness of the learning platforms used (Canvas and Zoom), the resources provided, and the program components (e.g., critiquing, skill development, and a career panel). They largely reported a strong understanding of the goals of different sections of a research proposal and how to integrate scientific literature and data into a proposal. However, they reported slightly lower scores in their confidence in applying the knowledge learned to specific proposal tasks (e.g., writing the Innovation section, estimating a sample size, and describing data collection/analysis). The lowest confidence score was associated with providing a critique of the Specific Aims page of a proposal. In the postprogram interview, scholars shared that written feedback was valued because it provided concise input and in a manner that they could return to as they continued to refine their proposal.

**Table 2:** India scholars’ assessment of mentored proposal development (Phase III)

Survey Statements	Average* *N* = 2
Knowledge of designing qualitative research proposals	
Understand how to integrate preliminary data to support my research proposal	4
Understand the goals of a Specific Aims page	4.5
Understand the goals of the study Innovation section	4.5
Understand how to write my research proposal for nonspecialized audiences	4.5
Understand the goals of the Significance section	5
Understand how to integrate scientific literature to support my research proposal	5
Understand how to develop a theoretical framework to support my research proposal	5
Understand the goals of the Research Approach	5
Understand the goals of the study timeline	5
Confidence in writing and critiquing sections of a qualitative research proposal	
Ability to critique a Specific Aims page	3.5
Ability to write the Innovation section	4
Ability to estimate an effective sample size	4
Ability to describe data collection procedures	4
Ability to describe data analysis procedures	4
Ability to write a Specific Aims page	4.5
Ability to write the Significance section	5
Ability to define the study population	5
Ability to develop a study timeline	5
Usefulness of program components and delivery^†^	
Canvas platform was effective in supporting my learning	4.5
Interactive Zoom sessions effectively supported my learning	5
Resource provided on Canvas (e.g., recorded lectures and examples) effectively supported my proposal development	5
Critiquing peers’ work was a useful learning strategy	5
The career panel session was beneficial for my long-term career goals	5
I was satisfied with the skills I learned in the program	5

* Average scholar scores measured on a scale of 1–5.

^†^ See the following associated evaluation comments from scholars’ in-depth interviews:

Usefulness of program components: “The perfect blend of lectures and discussion. The lectures were not too lengthy, this gave us a lot of time to discuss issues and ask questions.”

“I found the supplementary material (slides) to be most helpful in learning the flow of a proposal and its content. Learning from slides, followed by detailed feedback by mentors was very helpful.”

Program delivery: “I’m from India, and we’re used to our mentors and teachers being very strict. And I think they [QRL mentors] were really nice. I feel like they could have been even more direct.”

In Table 3, Tanzanian scholars reported that developing a workplan with their institutional supervisor was adequate for managing their work commitments, agreeing on protected time, and discussing strategies to manage challenges for scholars to participate in the program. Scholars also reported that program resources were very/extremely useful. They reported that both the synchronous and asynchronous feedback that they received from all mentors was more effective for developing their research proposal than for conducting their research. Scholars reported that one of most valuable aspects of in-country mentors was generating “research ideas that are local and meeting the national and international research priorities.” However, they also acknowledged the time pressure on in-country mentors, for example, “There is currently limited mentors who are interested, conversant and keen to provide mentorship in qualitative research. This makes the available mentors thinly spread among the mentees and hence limited time and support.” Scholars felt that the program could conduct regular check-ins with in-country mentors to discuss scholars’ progress, mentors’ challenges, and any additional support they need to mentor scholars. Overall, Tanzanian scholars reported that they were moderately prepared and confident to conduct a field study (e.g., submit ethical approval, train a field team, and collect data). They also felt moderately prepared to conduct leadership activities in qualitative research at their institutions, such as mentoring and engaging others in seminars and study groups on qualitative research. However, they reported lower confidence in analyzing qualitative data, which was not a core component of the program.

**Table 3:** Tanzania scholars’ assessment of mentored proposal development (Phase III)

Survey Statements	Average* *N* = 2
Effectiveness of asynchronous support^†^	
Feedback via e-mail from institutional mentors for developing research proposal	2.5
Feedback via e-mail from institutional mentors for conducting research project	2.5
Feedback via e-mail from Emory mentors for conducting research project	2.5
Feedback via e-mail from Emory mentors for developing research proposal	4
Effectiveness of synchronous support^†^	
Feedback during Zoom meetings with Emory mentors for conducting research project	2.5
Feedback during Zoom meetings with Emory mentors for developing research proposal	3.5
Feedback during calls/meetings with institutional mentors for developing research proposal	3
Feedback during calls/meetings with institutional mentors for conducting research project	3
Effectiveness of the program workplan	
For identifying potential challenges to program participation	2.5
For developing strategies to manage potential challenges to program participation	3
For agreeing on protected time with my supervisor	3
For developing a plan to manage my work commitments during the program	3.5
Preparedness to conduct qualitative research	
Submitting an institutional application for ethical approval	3.5
Training a field team for qualitative data collection	3.5
Collecting qualitative data for the research project	3.5
Mentoring others in my institution on qualitative research	3.5
Identifying resources to addressing training gaps for career development	3.5
Identifying opportunities to engage others in qualitative research	3.5
Confidence to conduct qualitative research	
Analyzing qualitative data	3
Mentoring others in my institution on qualitative research	3.5
Collecting qualitative data	3.5
Training a field team on qualitative data collection	3.5
Preparing a manuscript/conference abstract	3.5
Submitting an institutional application for ethical approval	4
Identifying funding opportunities	4
Usefulness of program components and resources	
“Further resources” module on the Canvas site (e.g., additional videos/reading)	3.5
QUAL-WORKS workshop on qualitative research	4
Tailored resources provided by mentors for the research project	4

* Average scholar scores measured on a scale of 1–5.

^†^ See the following associated evaluation comments from scholars’ in-depth interviews:

Effectiveness of asynchronous support: “The knowledge gained in qualitative methods was so useful from both the lectures and learning resources.”

Effectiveness of synchronous support: “The step-by-step review and discussion of the objectives and methodology section was of great value. It was refreshing to be able attain support and direction or guidance from a mentor after discussing the thought process in the formulation of the objectives and methodology. This was quite different from the general feedback or remote feedback that I would have otherwise received in an e-mail or in a group setting.”

“The virtual meetings to build up my proposal were an opportunity to directly engage with my mentors and gain clear response/feedback. The frequency of the meetings was so doable and allowed me to prepare and plan for a fruitful meeting beforehand.”

All scholars were also asked about suggestions for improving Phase III. They commonly reported that more time was needed to more fully engage with the resources provided and conduct deeper discussions on applying the methodology to their proposals. For example, “My only suggestion would be to have adequate time in between sessions and for the general program to be spread over a few more weeks,” and “I feel in general having a gap between sessions would’ve helped as I would’ve had more time to devote to one topic.”

The VIRDE program evaluation data collected by Vanderbilt University during Phase IV are anonymous, so data are not available for QRL scholars. However, during our postprogram interview, we asked scholars to reflect on their experiences of the VIRDE program. Scholars reported that they entered VIRDE with a better understanding of how to develop a competitive grant proposal than their peers as a direct result of participating in the QRL program. One scholar described only recognizing how strong their proposal was after seeing feedback provided to other VIRDE trainees, sharing, “And I saw the comments on some proposals of other people who were sharing it with me. There were a lot of suggestions. So, with me it just said, ‘oh, this is good,’ so I was happy about that. So that was actually thanks to the QRL sessions because in VIRDE there were 13 of us in the class and so, you don’t get that individual mentorship. And in these QRL sessions we discussed it to the death!” The preparation scholars received through the QRL program also gave them the confidence to support and mentor their peers on qualitative research while participating in VIRDE. For example, “Anything qualitative that I learned was from the workshops and QRL and it made me a lot more confident, that I went in there as a qualitative researcher…At VIRDE, I had a lot of discussions with my fellow scholars there, and I did help them out with any concerns they had or any questions they have with regards to qualitative. So, that was also because of QRL.” The scholars noted the effectiveness of sequencing the QRL components before entering VIRDE because the QRL program provided detailed mentoring on writing a proposal for qualitative research and VIRDE focused more on providing overarching proposal feedback and helping them understand the processes for applying for a US federal grant. As none of the VIRDE mentors had expertise in qualitative methods, at least one scholar found it difficult to integrate different feedback coming from multiple sources, including in-country mentors. This was described as a “roadblock” to finalizing and submitting the proposal they developed in the program.

#### Leadership in qualitative research.

The overarching goal of the QRL program was to facilitate leadership in qualitative research among public health professionals in LMIC institutions. For scholars who completed all program components (*N* = 2), we also assessed postprogram productivity and leadership activities in research, mentoring, and teaching on qualitative research through in-depth interviews and a brief survey 12 months after program completion. Table 4 lists scholars’ leadership achievements in qualitative research and related comments.

- **Research.** Scholars were highly productive in postprogram qualitative research activities, generating 30 research outputs. Furthermore, scholars reported that the QRL program gave them greater confidence in their qualitative research activities.
- **Mentoring.** Scholars provided extensive postprogram mentoring of 54 researchers (e.g. students, postdoctoral fellows, and research faculty) on qualitative research, both within and outside organizations. Mentoring activities involved advising on qualitative methodology, providing resources, assisting with proposal writing (e.g., qualitative objectives, methods section, and ethics), reviewing research instruments, and providing guidance on ethical approval. Scholars even advised others while participating in the VIRDE program (see the earlier section). These mentoring activities define scholars’ professional leadership in qualitative research after participating in the QRL program, whereby they are recognized as a key resource for qualitative research within their institution and externally.

A secondary goal of the QRL program was to create a supportive environment within scholars’ institutions whereby qualitative research may be better understood and valued, thus providing a more receptive environment for scholars’ own qualitative research. Including institutional colleagues in the Phase II workshop was intended to foster this supportive environment. Although workshop participation did create significant interest and demand for guidance on qualitative research among scholars’ institutional colleagues, scholars’ own promotion of qualitative research also led to their being recognized as key resources for qualitative research. Thus, the ability of QRL scholars to become key mentors on qualitative research within their institutions further enhanced the supportive environment for qualitative research.

- **Teaching.** Scholars developed and taught eight courses on qualitative research, which also attracted students from external organizations. This demonstrates an indirect ripple effect of the QRL program, whereby QRL scholars learn, teach, and gain broader recognition outside their institutions. Scholars also reflected on the added value of the QRL program in demonstrating how to teach qualitative research, and they described using similar materials, teaching techniques, and skill-building activities to develop and teach their own courses. Through teaching qualitative research, scholars also reported greater confidence in their own qualitative research abilities.

**Table 4:** Scholars’ leadership achievements in qualitative research

Research Achievements	Mentoring Achievements	Teaching Achievements
Five research proposals submitted Three research proposals funded Four research projects as collaborators Three research presentations (external conferences) Three international research presentations (e.g., China, Democratic Republic of the Congo, United States) Eight research seminars (internal) Four journal papers published “[QRL] affected my confidence positively, like a lot. It’s enabled me to teach others, to share resources with others, to write better, as a qualitative researcher. I have written an article recently, and I wrote it with a confidence…and that article ended up being published in a journal that has a high impact factor. So that of course, really reinforced my confidence positively. So, all that is connected to doing [QRL] and feeling confident.”	Forty-six researchers mentored within institution Eight researchers mentored externally “…a lot of people have reached out from my organization, from different departments, who are writing grants and who need help with the qualitative components. So, I have been able to proofread their guides, give some suggestions, suggest some readings as well, that would be helpful in writing methodology…so basically, I am known as the qualitative person in my organization.” “After the QRL skill training, a lot of colleagues were interested…I remember a lot of them were postgraduate students. So, a lot of them will come to say, I need help here, I need support. We had some books that [QRL Mentors] shared with us. They will come to us. Can I have a book? I want to review this particular topic. So, it really stimulated the interest to know more about qualitative methods.” “…even at VIRDE I had a lot of discussions with my fellow scholars there, and I did help them out with any concerns they had or any questions they have with regards to qualitative. So, that was also because of QRL.”	Six courses taught on qualitative research One short course taught (annually, internal) One training course (international) “When I do this qualitative method teaching, I use a lot of ideas and skills from [QRL Workshop]…the basic understanding of all these principles and approaches are very well grasped by myself, and even when I teach others.” “Myself and one of my colleagues, we took the opportunity to go to Congo and provide a 1-week training. So, we did really something similar to QRL training.” “Out of the few who are facilitating the course, I’m one of the top and I’m proud about it. And luckily, of course, through [QRL] training, I’ve been very confident in teaching others because I used similar techniques.” “The QRL Scholar Training Program has given me all the basics that are important for me to understand, and I have more confidence in even teaching others, mentoring others, in applying my own research grants I have been writing, I incorporate the aspect of qualitative and yeah, I think generally across all I feel confident.”

QRL = Qualitative Research Leaders; VIRDE = Vanderbilt Institute for Research Development and Ethics.

## DISCUSSION

Our evaluation results show that overall, the QRL program was effectively designed and delivered to meet its goals. Here, we reflect on some of the nuances of the evaluation results and lessons learned from implementing this pilot program.

### Program design.

The five-phase QRL program was effectively designed, and scholars valued the program components, learning formats, and session content. We found no difference in program delivery or evaluation results between the two countries in which the pilot program was implemented, which suggests that it could be delivered in a range of different global contexts and formats. However, for ease of delivery and minimal participant burden, we recommend selecting countries in similar time zones and developing alternative support strategies when internet connectivity is disrupted. One notable adjustment to the program design could be increasing the length of the program. Scholars suggested extending the entire program from 12 months to 18–24 months. This is not to add new components or content but to increase the time between each mentored session for scholars to apply the lessons learned to each section of their research proposals more fully, review readings and resources more deeply, and provide more time to reflect on learnings from each session. This change may improve scholars’ overall skills and confidence for proposal development. Extending the length of the program would necessitate providing scholars with more protected time away from their usual professional responsibilities and ensuring that program participation is an integral part of their work rather than an additional “after hours” activity. This could be established in scholars’ workplans developed in Phase I.

An important design element was delivering the 5-day workshop (Phase II) in person versus remotely. This provided an important touchpoint for connectivity between mentors and scholars. It enabled stronger rapport between mentors and scholars and allowed mentors to better understand the research environment of scholars in terms of infrastructure, resources, and their network of colleagues. Furthermore, the skill-building activities of the workshop also prompted richer discussions among participants when conducted in person. Additionally, to ensure scholars are committed to participating in all program components, eligibility screening criteria could be added that assess for concurrent participation in programs or fellowships that compete for scholars’ time and reduce their likelihood of program completion.

### Mentoring.

All mentoring components of the program were highly valued by scholars, including step-by-step development of their research proposal with individual feedback and tailored resources for each section of their proposal. They also valued receiving feedback in different formats—written, verbal, and peer-to-peer. However, we would recommend providing more written feedback, followed by discussion-based feedback to reinforce points. This allows scholars to refer to written comments, compensating for times when poor audio from internet connectivity issues interferes with verbal feedback. Evaluation results showed that mentoring was rated stronger for the proposal development phases than for conducting the study (e.g., data collection). The emphasis of the QRL program was for scholars to receive intensive mentoring during the proposal development stages of their project, after which they would be linked with mentors from a network of experts in their geographic region to receive advice as needed. In the future, we would recommend a two-phase mentoring plan, whereby scholars would still receive intensive structured mentoring from the QRL program for their proposal development but then fully transition to their in-country mentor for support during the fieldwork component of their project. In-country mentors are well placed to provide methodological advice that reflects the cultural context and fieldwork realities in the study country. Because in-country mentors are within the scholars’ institutions, they may also be more accessible for support when needed. Also, it would be challenging for the QRL program to provide intensive individual mentoring on scholars’ projects when funding timelines and fieldwork may proceed at a very different pace across scholars’ projects. This would become particularly challenging with program scale-up when more scholars would participate globally.

Scholars valued having an in-country mentor on the mentoring team to help them define local research issues to study and shape their proposal around national health priorities. However, scholars felt that there were few mentors in their institutions with expertise on qualitative research, which meant that these mentors often had limited time availability. We recommend being realistic about the time commitments of in-country mentors and providing support for them throughout the program,^9^ for example, by discussing protected time for mentors (not just scholars) to participate in the program, providing funding or resources to support their mentoring role, and allocating travel funds to attend external training on mentoring. Another useful addition could be to schedule review meetings with in-country mentors at specific time points throughout the program to provide touchpoints to review scholars’ progress and revisit support needed for in-country mentors.

### Scholar learning.

The evaluation results suggest that a cascade of learning occurred in the program, with the highest impact shown on scholars’ knowledge of qualitative research, then skill development and then confidence in mentoring others, which contributes to leadership in qualitative research. The greatest change in scholars’ knowledge was on the guiding principles for qualitative research (e.g., research paradigms and concepts), which are critical first-order skills needed before conducting, critiquing, or mentoring qualitative research. This change is important, as it indicates that scholars gained a deeper understanding of the guiding principles of qualitative research that determine how to define, conduct, and assess qualitative research. Understanding these concepts well is critical for embedding rigor into qualitative research. Next, an increase in skills was enabled through activity-based learning, which not only allowed scholars to apply their new knowledge but also increased their confidence to mentor others. Scholars showed the greatest confidence to mentor others on tasks that were part of a skill-building activity in the QRL sessions (e.g., determining a sample size). This underscores the importance of incorporating skill-building activities in qualitative research training, not only to increase knowledge and skills but also to foster confidence needed to mentor others and become leaders in qualitative research. Therefore, we suggest incorporating more activities to increase scholars’ confidence with qualitative research and thereby increase their confidence to effectively mentor others. Confidence-building activities may simply include more skills-based activities, for example, more peer critiques and more case studies to practice critiquing specific sections of a study proposal (e.g., study objectives, Significance and Innovation sections, and study design). Finally, scholars’ confidence for critiquing and mentoring others was lower than the knowledge and skills gained in the program. This ordering is understandable, given that critiquing and mentoring are higher-order skills that develop after knowledge and skills are acquired. Overall, acknowledging a sequenced process of learning and allowing time for this is important for the program. By extending the program (as discussed above), scholars may have more time to absorb the knowledge gained more fully and more time for application and thus skill development, and both may then increase scholars’ confidence in mentoring others on qualitative research. This would increase the program’s overall impact for developing scholars into effective mentors and leaders on qualitative research.

## CONCLUSION

The QRL program provides a framework for an effective training pipeline to address critical shortages in qualitative research expertise in LMIC institutions. The program design, content, and delivery were favorably evaluated by scholars, and program fidelity suggests that it can be implemented in diverse global settings. Further adjustments could include extending the program length to increase the overall impact on scholars’ learning and confidence, thereby generating more effective mentors and leaders in qualitative research within LMIC institutions.

## Supplemental Materials

10.4269/ajtmh.24-0381 Supplemental Materials
