# Supplementary material for: Qualitative Research Leaders: Evaluation of Pilot Global Pipeline Program
Source: Am J Trop Med Hyg. 2024 Dec 10;112(3):699–708. doi: 10.4269/ajtmh.24-0381 (PMC11884274; doi:10.4269/ajtmh.24-0381)
Supplement: Supplemental Materials [file tpmd240381.SD1.pdf]

Supplementary Figure 1. Scholar's Assessment of Study Design Sessions (Phase I)

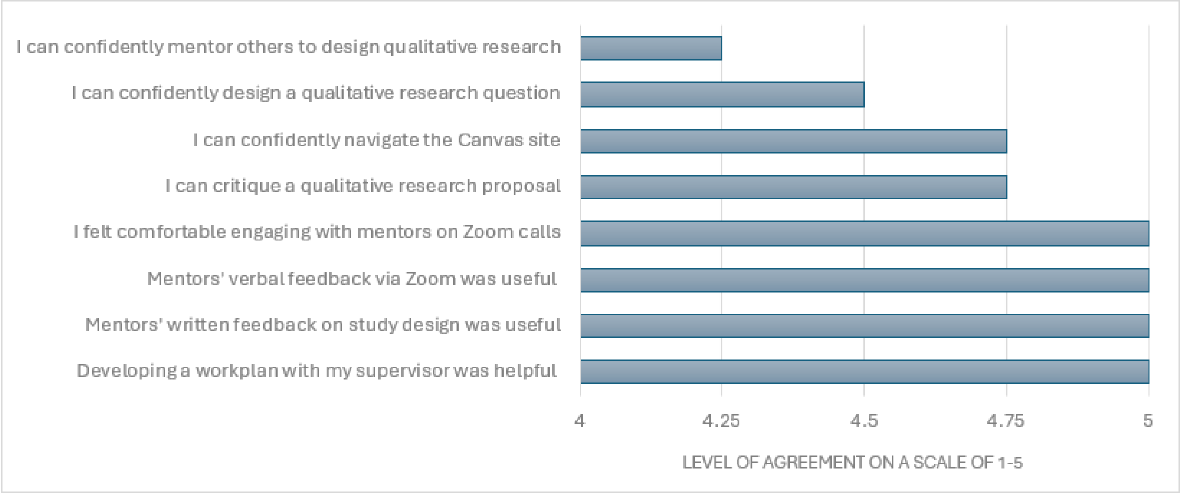

Note: n=4 [including the QRL scholars from India (n=2) and Tanzania (n=2)].
